# Evaluation of the Antioxidant and Anti-glication Effects of the Hexane Extract from *Piper auritum* Leaves *in Vitro* and Beneficial Activity on Oxidative Stress and Advanced Glycation End-Product-Mediated Renal Injury in Streptozotocin-Treated Diabetic Rats

**DOI:** 10.3390/molecules171011897

**Published:** 2012-10-09

**Authors:** Rosa Martha Perez Gutierrez, Luis B. Flores Cotera, Adriana Maria Neira Gonzalez

**Affiliations:** 1 Laboratorio de Investigación de Productos Naturales, Escuela Superior de Ingenieria Quimica e Industrias Extractivas IPN, Av. Instituto Politécnico Nacional S/N, Unidad Profesional Adolfo Lopez Mateos, cp. 07708, Mexico D.F., Mexico; 2 Laboratorio de Metabolitos Secundarios de Microorganismos, Departamento de Biotecnologia y Bioingenieria, Cinvestav, Av. no. 2508, cp. 07360, Mexico D.F., Mexico

**Keywords:** *Piper auritum*, antioxidant, antidiabetic, antiglycation

## Abstract

The aim of this study was to investigate the antioxidant activity of hexane extracts from leaves of *Piper auritum* (HS). Eight complementary *in vitro* test methods were used, including inhibition of DPPH· radicals, nitric oxide, superoxide anion, ion-chelating, ABTS, oxygen radical absorbance capacity, β-carotene bleaching and peroxy radical scavenging. The results indicated that HS possesses high antioxidant activity. To add to these finding we tested the effect against oxidative stress in liver, pancreas and kidney in diabetic rats. Low levels of SOD, CAT, GPx and GSH in diabetic rats were reverted to near normal values after treatment with HS. These results suggest that *P. auritum* prevents oxidative stress, acting as a suppressor of liver cell damage. Given the link between glycation and oxidation, we proposed that HS might possess significant *in vitro* antiglycation activity. Our data confirmed the inhibitory effect of HS on bovine serum albumin, serum glycosylated protein, glycation of LDL, and glycation hemoglobin. The effect of HS on diabetic renal damage was investigated using streptozotocin-induced diabetic rats. The oral administration of HS at a dose of 200 and 400 mg/kg body weight/day for 28 days significantly reduced advanced glycation endproduct (AGE) formation, elevated renal glucose and thiobarbituric acid-reactive substance levels in the kidneys of diabetic rats. This implies that HS would alleviate the oxidative stress under diabetes through the inhibition of lipid peroxidation. These findings indicate that oxidative stress is increased in the diabetic rat kidney and that HS can prevent renal damage associated with diabetes by attenuating the oxidative stress.

## 1. Introduction

Reactive oxygen species (ROS) are generated in all living organisms mainly during mitochondrial metabolism [1]. ROS may include superoxide anion (O_2_**^.^**^−^), hydroxyl radical (OH**^.^**), hydrogen peroxide (H_2_O_2_) and nitric oxide (NO). Excessive amounts of ROS may overwhelm natural antioxidant defenses promoting DNA, lipid and protein oxidative damage and oxidative stress, which may lead to cell injury and death [2]. Long term oxidative stress has been associated with numerous diseases and disorders in higher organisms. The negative effects of oxidative stress may be mitigated by the consumption of antioxidants. Antioxidants are compounds that can delay or inhibit the oxidation of lipids and other molecules, and by doing so inhibit the initiation and propagation of oxidative chain reactions. They act by one or more of the following mechanisms: reducing activity, free radical-scavenging, potential complexation of pro-oxidant metals and quenching of singlet oxygen. Many epidemiological studies have shown that numerous phytonutrients found in fruits and vegetables are able protect the human body against damage by ROS. In fact it is well established that the consumption of natural antioxidant phytochemicals was reported to have many potential health benefits [3]. In recent years, there has been a renewed interest in finding natural antioxidants from plant materials.

Many researchers have discussed the pathological features of diabetes and its complications, which are caused to a great extent by the accelerated formation of advanced glycation endproducts (AGEs) promoted by hyperglycemia in tissues [4]. Inhibition of AGE formation has been an effective way for retarding the full range of diabetes complications, such as nephropathy, neuropathy, retinopathy, and vasculopathy.

The large-leafed, perennial plant *Piper auritum*, known as ‘Hoja Santa’, is used in México as a spicy aromatic scent and flavor, and for its therapeutic properties. It has been used traditionally as an emollient, antirheumatic, diuretic, stimulant and abortifacient, anti-inflammatory, antibacterial, antifungal and antidermatophytic [5]. The main components of the essential oil are safrol, and myristycin [3]. Navarro *et al*. [6] reported that methanol and aqueous extracts from *P. auritum* had no activity against DPPH, O_2_**^−^** and ^.^OH. Our aim was to study *in vitro* and *in vivo*, the antioxidant activity and the inhibition of AGEs formation of the hexane extract of *P. auritum* leaves. 

## 2. Results and Discussion

### 2.1. Total Phenolic Content

The total phenolic content in HS in leaves was rather low, *i.e.*, 0.3% (w/w), so the strong antioxidant activity of the hexane extract appears to be related to its content of hydrophobic compounds, therefore, there is no correlation with total phenolic content in HS.

### 2.2. Antioxidant Activity *in Vitro*

#### 2.2.1. 1,1-Diphenyl-2-picrylhydrazyl (DPPH) Assay

**Figure 1:**
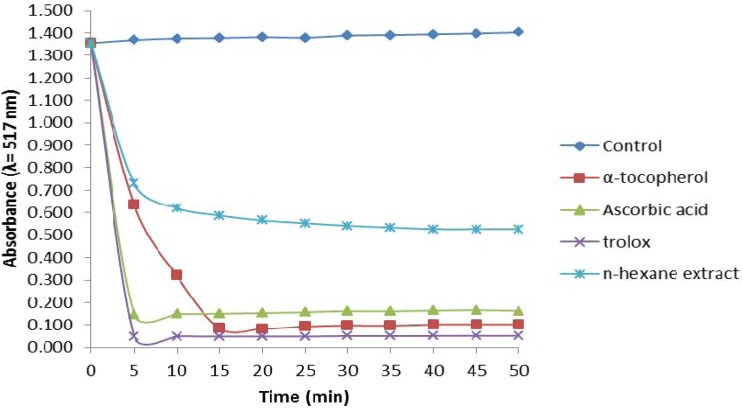
DPPH scavenging activity of the *n*-hexane extract of the leaves of *P. auritum*, compared to α-tocopherol, ascorbic acid and Trolox. Values are mean ± SEM and significantly *p* < 0.05.

DPPH· is a stable free radical that is able to accept one electron or hydrogen atom to form a stable diamagnetic molecule. The reaction is visually noticeable as a discoloration from purple to yellow. Antioxidants react with a number of DPPH· molecules equivalent to the number of their available hydroxyl groups. Therefore, the absorbance at 517 nm is proportional to the amount of residual DPPH·. Antioxidants can be classified, according to the time needed to reach a steady state absorbance as follows: rapid <5 min, intermediate 5–30 min and slow >30 min [7]. Figure 1 illustrates the kinetic behaviour of hexane extract as radical scavenger toward DPPH. A step fall in absorbance occurred in the assay with HS within the first five min, but after 10 min the absorbance decreased slowly. Thus, the HS antioxidant activity can be considered intermediate since a steady state after 30 min is usually considered low activity [8]. The three control antioxidants reacted faster with the DPPH· radical than with HS. Ascorbic acid and Trolox reached steady states within five min, whereas the absorbance with α-tocopherol was relatively constant after 15 min (Figure 1). Our results agree with previously reported data which considered that the kinetic classification of the former compounds was rapid and that of α-tocopherol was intermediate [8].

#### 2.2.2. Trolox Equivalent Antioxidant Capacity (TEAC) Assay

**Figure 2:**
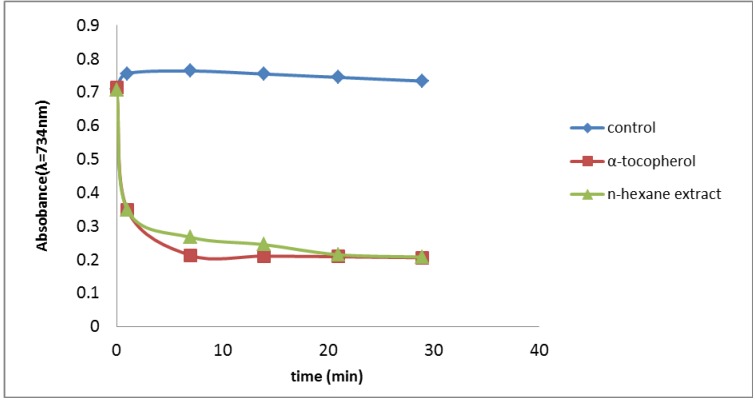
ABTS scavenging activity of the *n*-hexane extract of the leaves of *P. auritum* and α-tocoferol. Values are mean ± SEM and significantly *p* < 0.05.

Oxidation of ABTS with potassium persulfate (with absorption maxima at 734 nm) leads to the generation of ABTS free radicals. This method is based on the ability of antioxidants to quench the ABTS^+^ radical cation [7]. Figure 2 shows the time course of absorbance during the assay with HS, where we found that 30 min of inhibition was required for complete suppression of ABTS radical formation in the TEAC assay (Figure 2). The percentage inhibition was 50.8% and 70.5% for the HS and α-tocopherol, respectively, at 0.1 mg/mL each. The HS is thus an effective scavenger of the ABTS radical with antioxidant activity comparable to Trolox.

#### 2.2.3. Oxygen Radical Absorbance Capacity (ORAC) Assay

**Figure 3:**
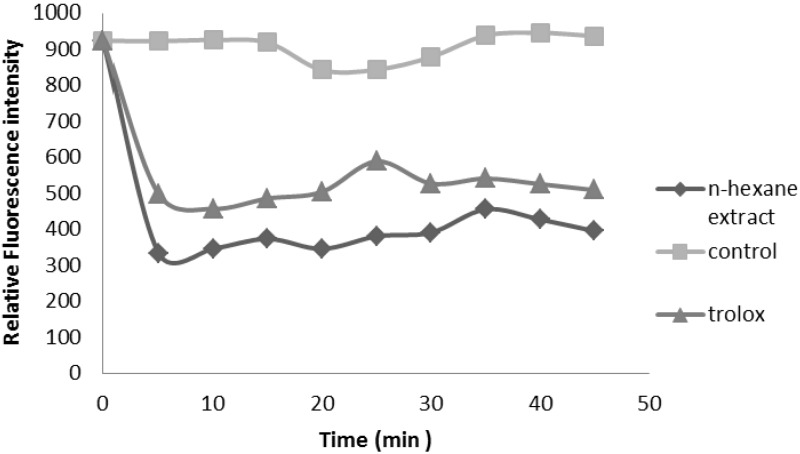
Illustration of calculation the ORAC value expressed as the net area under the curve (AUC).

The peroxyl radical scavenging activity of the extracts was determined by an ORAC assay. This assay is based on the susceptibility of sodium fluorescein to AAPH, with concomitant loss of its fluorescence [9]. HS showed significant antioxidant potential, and the values represent ORAC_ROO+_ activities of the tested extracts equivalent to Trolox. The results showed HS with the ORAC_ROO+_ value of 48.3 ± 4.28 µmol TE/g DM (Figure 3).

The correlation of the two assays (ORAC and TEAC) was not high. A poor correlation may be expected because different free radical sources are used in the two methods. The TEAC assay uses exogenous ABTS radicals, whereas the ORAC assay uses peroxyl radicals. Because peroxyl radicals are the most common radicals found in the human body, ORAC measurements should be more biologically relevant. Both assays are inhibition methods: TEAC reflects the relative ability of hydrogen or electron donating antioxidants to scavenge the ABTS radical cation compared with Trolox while ORAC is a method to measure the scavenging activity of peroxyl radicals. The ORAC, ABTS, and DPPH values indicated that HS possesses significant radical quenching property.

#### 2.2.4. Ferrous Ion Chelating Ability

**Figure 4:**
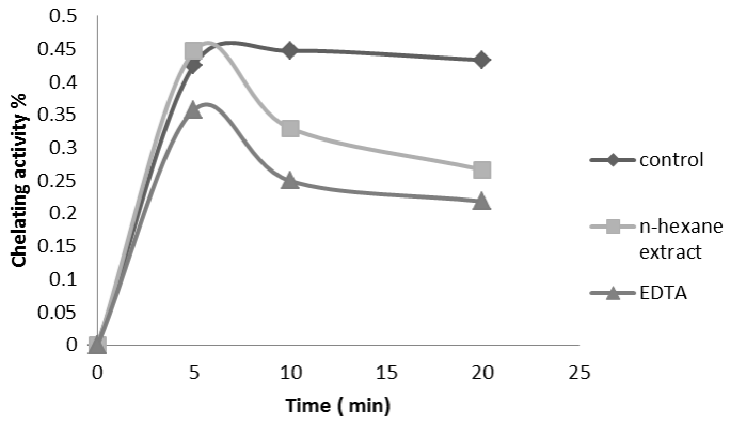
Formation of the Fe^2+^-ferrozine complex of *n*-hexane extract of the leaves of *P.auritum* and EDTA. Values are mean ± SEM and significantly *p* < 0.05.

Iron is the most important lipid pro-oxidant. It is known that Fe^2+^-accelerates lipid peroxidation by breaking down hydrogen and forming lipid peroxides by Fenton free radical reactions [10]. As shown in Figure 4, the formation of the Fe^2+^-ferrozine complex was incomplete in the presence of *P. auritum* hexane extract, suggesting that HS was able to chelate the metal. The difference between both the extract and the control was statistically significant (*p* < 0.05). The Fe^2+^ chelating capacities of HS and EDTA were 37.32%, and 38.59%, respectively. Both the hexane extract and the standard antioxidant (EDTA) compete for the metal with ferrozine, suggesting that they have chelating activity, capturing the ferrous ion before it can form a complex with ferrozine, showing that the kinetic for HS and EDTA was stable at 20 min.

#### 2.2.5. Nitric Oxide Radical Scavenging Assay

**Figure 5:**
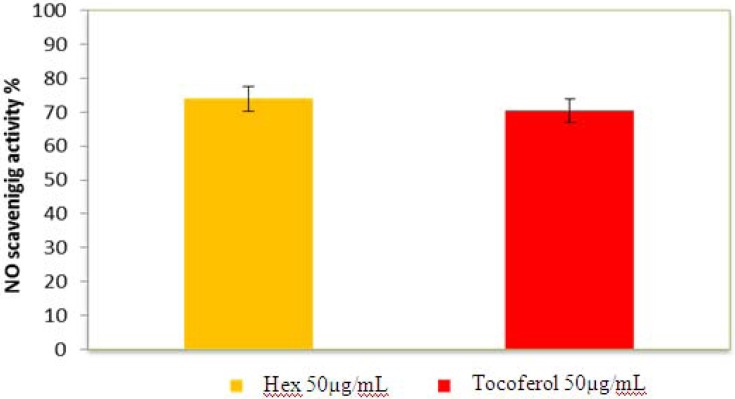
Nitric oxide radical scavenging activity of HS and α-tocoferol. Values are mean ± SEM and significantly *p* < 0.05.

Nitric oxide (NO) is a particularly oxygen reactive species. Although its excess can produce harmful effect in the organisms, it is also a very important cell mediator that regulates a number of functions and cell process in the organism. For instance, nitric oxide possesses vasodilatory, anti-inflammatory, anti-proliferative, cardiovascular anti-geratogenic and anti-platelet activities [11] Thus, the present results show that hexane extract may be helpful in the management of many diseases that require NO such as arterial hypertension with associated endothelial dysfunction. Figure 5 illustrates decrease in the concentration of nitric oxide free radicals due to the scavenging ability of *P.* *auritum* and standard. A 50 µg/ mL of *P.* *auritum* and ascorbic acid (std.) exhibited 74% and 70.45% inhibition, respectively. The NO scavenging capacity of extract was higher than that of quercetin. This may be due to the antioxidant properties in the extract which compete with oxygen to react with nitric oxide thereby inhibiting the generation of peroxynitrite (ONOO^−^).

#### 2.2.6. β-Carotene Bleaching (BCB) Assay

**Figure 6:**
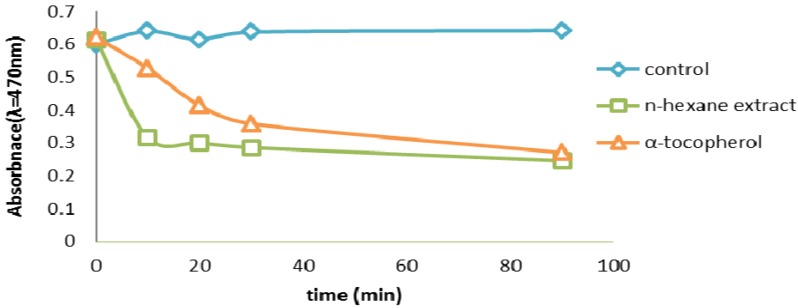
β-carotene scavenging activity (BCB) assay of HS and α-tocoferol. Values are mean ± SEM and significantly *p* < 0.05.

The β-carotene bleaching test method employs an emulsified lipid which introduces an enhanced number of variables influencing oxidation such as the partition of the compounds between the water and the lipid phase [12]. The more apolar a compound is, the stronger is its because it concentrates at the lipid-air surface, thus ensuring high protection of the emulsion itself. Our results showed that the methanol extract which is the most polar is the less active that the hexane extract which is the most apolar exhibited the strongest antioxidant activity. HS exhibited the highest antioxidant activity, with 79% of β-carotene remaining after 20 min (Figure 6). The β-carotene bleaching test is similar to an oil-in-water emulsion system; differences in the solubility’s of antioxidant compounds influence their activity in this assay, Hydrophobic antioxidants are reported to perform more efficiently than hydrophilic antioxidants in the β-carotene bleaching test, this because has affinity for the lipid phase, where they can react with peroxyl radicals avoiding β-carotene oxidation [9]. The strong activity of HS components may be due to their higher level of hydrophobic antioxidants.

#### 2.2.7. Thiocyanate Method

**Figure 7:**
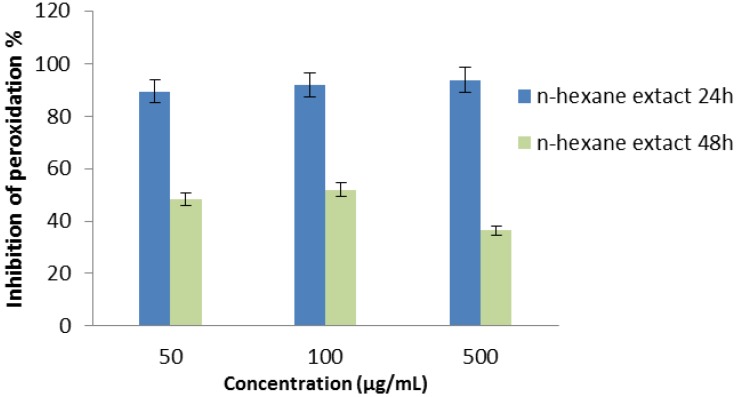
Inhibition of linoleic acid peroxidation of the hexane extract of the leaves of *P.auritum* determined by ferric thiocyanate assay.

Figure 7 shows that the hexane extract of *P. auritum* inhibited linoleic acid peroxidation. In this system, ferrous ion is oxidized by linoleate radicals such as hydroperoxides to form ferric ion which is monitored as a thiocyanate complex at 500 nm. The antioxidant compounds in the plant extract prevent oxidation of ferrous ion inhibiting the linoleate radicals in system. In this system, oxidation of linoleic acid was effectively inhibited by extract of HS. The hexanic extract of HS showed high antioxidant activity with 78.3 ± 1.7% by inhibiting the formation of ferric ion.

#### 2.2.8. Superoxide Radical Scavenging Assay

**Figure 8:**
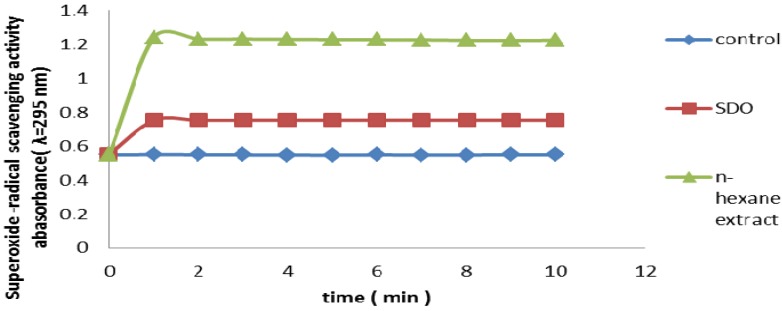
Superoxide radical scavenging activity of hexane extract from leaves of *Piper Auritum.* α-tocopherol was used as reference antioxidant and SOD as control. Values are mean ± SEM. (n = 3).

Superoxide anion is a precursor to the formation in living systems of other ROS such as hydroxyl radical, hydrogen peroxide, or singlet oxygen. Therefore, superoxide anion scavenging is important for antioxidant activity. The production of highly reactive oxygen species such as superoxide anion radicals is catalyzed by free iron through the Haber-Weiss reactions [13] which is a reduced form of O_2_^.^ has been implicated in the initiating oxidation reactions associated with aging. In the PMS/NADH-NBT system, superoxide anion derived from dissolved oxygen by PMS/NADH coupling reaction reduces NBT. Antioxidants are able to inhibit the blue NBT formation. The decrease of absorbance at 560 nm with antioxidants thus indicates the consumption of superoxide anion in the reaction mixture, Figure 8 presents the superoxide radical scavenging activity of 100 µg/mL HS in comparison with the same dose of α-tocopherol, Hexane extract had a superoxide radical scavenging activity, less than that of reference antioxidant at the same concentrations, (*p* < 0.05).

### 2.3. Antioxidant Activity *in Vivo*

#### 2.3.1. Measurements of GSH, SOD, CAT and GPx

The antioxidant effect of the *P. auritum* extract on tissue oxidative markers was studied here (Table 1). Diabetic rats showed a significant reduction in SOD, CAT, GSH and GPx in hepatic, pancreatic and renal tissues. Low levels of SOD, CAT, GSH and GPx in diabetic rats were reverted to near normal values after treatment with hexane extract. The readings obtained from the treated groups were comparable to that of the standard drug glibenclamide. Administration of *P. auritum* to diabetic rats showed restoration of liver, pancreas and kidney activities as reflected by these parameters. Hyperglycemia-induced oxidative stress may also cause liver cell damage. Lower activity of antioxidant enzymes such as SOD, GSH, GPx, CAT and increased rate of glycation oxidation leads to diabetic complications. Levels of these enzymes were back to near normal values after treatment with *P. auritum* extract. These results suggest that *P. auritum* prevents oxidative stress, acts as a suppressor of liver cell damage and inhibits the progression of liver dysfunction induced by chronic hyperglycemia. In total, these results suggest that hepatoprotection shown by the *P.* *auritum* extract may be due to its antioxidant properties.

**Table 1:** Changes of superoxide dismutase (SOD), catalase (CAT), glutathione peroxidase (GPx), and glutathione reductase (GSH) in liver, pancreas and kidney in diabetic rats treated with HS.

Parameters	Normal Control	Diabetic control	Diabetic+HS (200 mg/kg)	Diabetic+HS (400 mg/kg)	Diabetic+ GB (5 mg/kg)
SOD-Liver	7.65 ± 2.54	3.29 ± 1.57 ^a^	5.87 ± 1.29 ^c^	6.41 ± 1.79 ^b^	6.82 ± 2.18 ^c^
SOD-Kidney	14.03 ± 0.36	8.04 ± 2.49 ^a^	10.47 ± 1.37 ^b^	12.19 ± 2.58 ^b^	12.97 ± 1.26 ^b^
SOD-Pancreas	54.1 ± 3.54	35.76 ± 5.15	39.36 ± 3.42 ^b^	47.59 ± 3.52 ^c^	51.78 ± 4.77 ^b^
CAT-Liver	82.10 ± 1.79	43.35 ± 4.94 ^a^	61.25 ± 3.47 ^c^	70.63 ± 2.80 ^b^	74.67 ± 5.49 ^b^
CAT-Kidney	34.85 ± 2.65	21.49 ± 1.58 ^a^	30.27 ± 4.43 ^b^	33.90 ± 2.60 ^c^	34.32 ± 1.76 ^b^
CAT-Pancreas	59.6 ± 3.17	25.41 ± 3.12 ^a^	35.47 ± 2.80 ^c^	49.67 ± 4.21 ^c^	51.29 ± 4.48 ^c^
GSH-Liver	46.48 ± 2.34	23.72 ± 1.80 ^a^	37.17 ± 4.54 ^b^	43.26 ± 1.73 ^b^	42.87 ± 3.31 ^b^
GSH-Kidney	24.11 ± 0.73	5.78 ± 0.84 ^a^	16.73 ± 2.41 ^b^	20.21 ± 2.68 ^b^	19.86 ± 1.13 ^b^
GSH-Pancreas	11.9 ± 1.23	6.58 ± 0.91 ^a^	8.09 ± 2.39 ^c^	10.26 ± 1.87 ^c^	10.98 ± 1.54 ^c^
GPx-Liver	7.43 ± 2.17	4.56 ± 0.24 ^a^	5.69 ± 1.52 ^c^	6.23 ± 1.27 ^b^	5.90 ± 0.75 ^b^
GPx-Kidney	5.89 ± 0.78	3.49 ± 0.18 ^a^	4.11 ± 1.36 ^b^	4.67 ± 0.17 ^b^	4.53 ± 0.90 ^b^
GPx-Pancreas	4.12 ± 1.09	2.18 ± 0.63 ^a^	2.98 ± 0.53 ^c^	3.67 ± 0.82 ^b^	3.89 ± 0.68 ^c^

All values are expressed as Means ± SEM, n = 6 Values. ^a^ *p* < 0.01 when compared to normal control group; ^b^ *p* < 0.01 and ^c^ *p* < 0.05 compared to diabetic control group; where the significance was performed by Oneway ANOVA followed by *post hoc* Dunnett’s test. Glibenclamide (GB). The values are given in U/mg of protein.

#### 2.3.2. TBARS Levels in the Liver and Pancreas

There was a significant elevation in TBARS levels in the liver and pancreas of diabetic rats (Table 2). Daily administration of *P. auritum* extracts at a dose of 400 mg/kg to diabetic rats for 28 days significantly reduced TBARS levels by 45.4% in liver and 48% in pancreas. STZ induction of diabetes in rats leads to lipid peroxidation. TBARS are an index of endogenous lipid peroxidation and oxidative stress as an intensified free radical production [14]. Increased TBARS suggests an increase in oxygen radicals that could be caused either by an increased production or by a decreased destruction. It is well known that insulin activates lipoprotein lipase, which hydrolyzes triglycerides under normal conditions. Destruction of β-cells leads to depletion of plasma insulin, producing a derangement of metabolic pathways causing abnormalities that lead to hyperlipidemia and hypercholesterolemia [15]. These elevated TBARS levels in diabetic rats might be due to the stimulation of hepatic triglyceride synthesis as a result of free fatty acid influx [16]. We proved that repeated administration of *P.* *auritum* has a beneficial effect lowering hyperlipidemia associated with hyperglycemia, so we assumed that HS might mitigate the oxidative stress in liver, pancreas and kidney in STZ-induced diabetic rat.

**Table 2:** Changes in the contents of TBA-reactive substance in liver and pancreas in diabetic rats treated with HS.

Group	Liver	Pancreas	
Normal control	1.15 ± 0.35		0.484 ± 0.001
Diabetic control	2.18 ± 0.61 ^a^		2.87 ± 0.054 ^a^
Diabetic + HS (200 mg/kg)	1.73 ± 0. 12 ^b^		1.62 ± 0.061 ^b^
Diabetic+ HS (400 mg/kg)	1.19 ± 0.23 ^b^		1.49 ± 0.036 ^b^
Diabetic+ GB (0.5 mg/kg)	1.03 ± 0. 40 ^b^		1.22 ± 0.029 ^b^

All values are expressed as Means ± SEM, n = 6 Values. ^a^ *p* < 0.01 when compared to normal control group; ^b^ *p* < 0.01 compared to diabetic control group; Glibenclamide (GB). The values are given in mmol/mg of protein.

Due to the complex nature of extracts derived from *P. auritum*, it is insufficient to evaluate the antioxidant activity only with a single method. Therefore, several method were performed, to determine the abilities of the extracts to inhibit oxidation, which are related to the type of radical generated and to the polarity of the substrate system, and therefore, hard to determine. HS showed higher antioxidant effects *in vitro* as compared with the methanol and chloroform extracts *P. auritum* leaves, which produce only small effect so the results are not presented here. 

One of the most important points for an antioxidant substance is, besides being a good antioxidant *in vitro*, also being able to reach the site of action in its active form and act on easily oxidizable physiological substrates *in vivo.* In this study, HS exhibited significant free radical scavenging activities and anti-lipid peroxidation effects *in vitro* and we also have proved that oral administration of HS could decrease the liver, pancreas and kidney lipid peroxide levels in rats. These three organs are commonly and easily susceptible to oxidative stress. On the other hand, liver is metabolically active postmitotic tissues, so it is easy to generate free radicals by drug metabolising system. Many researchers have reported that the accumulation of lipid peroxide and unsaturated fatty acid in liver are associated with acute and chronic liver injury [17], so HS could be effective on the organs where free radicals are easily generated and which contain a high level of unsaturated fatty acid targeted by the free radicals.

### 2.4. *In Vitro* Glycation of Proteins

#### 2.4.1. BSA-Glucose and BSA-Methylglyoxal Assays

In order to determine the inhibitory effect of the hexane extract from *P. auritum* on AGEs formation, several assay methods have been proposed, including assays based on inhibition of specific fluorescence generated during the course of glycation and AGEs formation, and assays based on the inhibition of AGEs-protein cross-linking. HS, phloglucinol and aminoguanidine exhibited higher inhibitory activity against AGEs formation after incubation at 37 °C for 15 days, with an IC_50_ value of 0.420, 0.070 and 0.323 mg/mL respectively (Table 3). Methylglyoxal-mediated protein glycation inhibition was evaluated for HS which exhibited a substantial activity, compared with phloglucinol and aminoguanidine (Table 3), with IC_50_ values of 0.286, 0.060 and 0.195 mg/mL, respectively. In this study we found that HS inhibited formation of methylglyoxal-derived advanced glycation end-products in a bovine serum-albumin-methylglyoxal system, and may also act by blocking conversion of dicarbonyl intermediates to advanced glycation endproducts. Dicarbonyl intermediates such as methylglyoxal have received considerable attention as mediators of advanced glycation endproduct formation and are known to react with lysine, arginine and cysteine residues in proteins to form glycosylamine protein crosslinks [18]. 

**Table 3:** Inhibitory effects of hexane extract from *Piper auritum* (HS) and aminoguanidine on the formation of advanced glycation end products (AGEs), *in vitro* induced byglucose and methylglioxal.

Inducer	Treatment	AGEs IC_50_ (mg/mL)
Glucose	Hexane extract (HS)	0.420 ± 0.062
	Aminoguanidine	0.323 ± 0.081
	Phloroglucinol	0.070 ± 0.0049
Methylglyoxal	Hexane extract (HS)	0.286 ± 0.039
	Aminoguanidine	0.195 ± 0.021
	Phloroglucinol	0.060 ± 0.0072

Data are mean ± standard deviation of triplicate tests.

#### 2.4.2. Glycation of Hemoglobin

Table 4 shows the amount of glycated hemoglobin (% GHb). When hemoglobin was used alone (NC), the amount of glycated hemoglobin was 9.5%. This noticeably increased with the addition of glucose to a 27.6% (PC). Nonetheless, it decrease significantly with the treatment of HS (18.6%) and dropped further with the treatment of glutathione (8.1%). The amount of hemoglobin A_1c _(%HbA_1c_), corresponds to a specific sub-fraction of glycated hemoglobin, it is lower than the amount of glycated hemoglobin, however, it showed a similar tendency in the percentage of glycation. This result indicates that HS produces the most potent glycation inhibition at the early stage of protein glycation at a concentration of 10 mg/mL. This plant therefore can effectively prevent HbA_1C_ formation. In the present study, typical characteristics of diabetes were shown. First is the increase of serum glycosylated protein, which is a parameter caused by glucose and other reducing sugars such as ribose and fructose reacting with the amino residues of proteins to form Amadori products, for instance, glycosylated hemoglobin (HbA_1c_) and the O_2_^−^ is also generated in the process of AGE formation. HS could directly decrease the formation of glycated hemoglobin, possibly as a result of the antioxidative activity [19,20].

#### 2.4.3. *In Vitro* Glycation of LDL

Amphotericin B treatments at 5 and 10 µM significantly increased LDL oxidation levels as determined by MDA formation. However, the presence of the extract at 5 and 10 µg/mL significantly reduced 10 µM amphotericin B-induced LDL oxidation (Table 5, *p* < 0.05). 

**Table 4:** Effects of administration of hexane extract of HS on glycosylated protein and glycated, hemoglobin GHb and HbA_1c_.

Groups	GHb	HbA_1c_	Glycosylated protein
Negative Control	8.9 ± 0.06	7.9 ± 0.98	15.3 ± 1.47
Positive control	27.6 ± 1.34	17.5 ± 1.56	23.7 ± 2.19
Methanol extract	18.6 ± 1.53 ^a^	14.9 ± 1.25 ^a^	19.1 ± 2.04 ^a^
Glutathione	8.1 ± 0.08 ^a^	9.0 ± 0.67 ^a^	-
Aminoguanidine	-	-	20.2 ± 1.87 ^a^

Negative control: Incubation with hemoglobin (30 mg/mL); positive control: Incubation with hemoglobin (30 mg/mL) + glucose (0.278 mM); hexane extract: Incubation with hemoglobin (30 mg/ dL) + glucose (0.278 mM) + hexane extract (10 mg/ mL); glutathione: Incubation with hemoglobin (3 mg/ dl) + glucose (2.7 mM) + glutathion (0.5 mM). Data as expressed as ± SD; ^a^ *p* < 0.05 *vs*. positive control values. Value as expressed in nmol/mg protein.

**Table 5:** The prooxidant effect of amphotericin B (AB) at 5 and 10 µM, and the antioxidant protection of 5 µM HS against 10 µM, AB-induced malondialdehyde (MDA) formation after a 48-h incubation at 37 °C.

Groups	MDA formation (nmol/mg LDL protein)
Control	13.40 ± 1.58
AB 5 µM	22.25 ± 4.39 ^a^
AB 10 µM	34.27 ± 2.91 ^a^
AB 10 µM + HS (5 µM)	28.11 ± 3.89 ^a^

Values are expressed as Mean ± SD, ^a^ Significantly (*p* < 0.05) different from control, where the significance was performed by Oneway ANOVA followed by *post hoc* Dunnett’s test. Value as expressed in nmol/mg LDL protein.

LDL treated with 50 mM glucose significantly increased glycation level (Table 6, *p* < 0.05). Under EDTA protection, the presence of the HS 5 and 10 µg/mL significantly reduced LDL glycation. On the other hand, both LDL oxidation and LDL glycation significantly increased when LDL was treated with 50 mM glucose without EDTA protection. The presence of HS at 5 and 10 µg/mL significantly reduced both LDL oxidation and glycation when compared with controls *( p* < 0.05), acting as antioxidative and antiglycative agent. 

**Table 6:** Protective effect of 5 µM, of HS on LDL against 50 mM glucose-induced glycation and oxidation with or without 0.5 mM EDTA treatment.

Treatment	With EDTA		Without EDTA	
	Glycation	Oxidation	Glycation	Oxidation
LDL	2.9 ± 0.076	3.4 ± 0.51	3.7 ± 1.02	21.49 ± 2.62
LDL+glucose	17.6 ± 2.94 ^a^	4.8 ± 1.64 ^a^	22.83 ± 2.35 ^a^	58.761 ± 3.29 ^a^
HS (5 µM)	6.39 ± 1.39 ^a,b^	3.2 ± 0.46 ^b^	15.27 ± 0.95 ^a,b^	46.50 ± 4.30 ^a,b^

Values are expressed as Means ± SD (n = 6), ^a^ Significantly (*p* < 0.05) different from LDL group. ^b^ Significantly (*p* < 0.05) different from LDL+glucose, where the significance was performed by Oneway ANOVA followed by *post hoc* Dunnett’s test.

The next is abnormal lipid metabolism, which can lead to lipid peroxidation with reactive oxygen species (ROS) and renal lipid accumulation, playing a role in the pathogenesis of diabetic nephropathy. Nonenzymatic glycation of LDL, is accompanied by oxidative, radical-generating reactions. In the presence of EDTA, oxidation was not responsible for the observed glycation; in this instance, glycation may be due simply to the interaction between LDL protein and glucose. On the other hand, elevated levels of LDL glycation were observed when LDL oxidation was not suppressed. In this condition, the oxidation from LDL should be an important contributor toward the elevated glycation because HS, was a powerful antioxidative and antiglycative agent. That is, HS might first retard the oxidation that occurred in LDL lipid, and then retard the subsequent oxidation-related glycation. Such results bear out that LDL glycation is strongly related to its oxidation [21], and also support the idea that delaying LDL oxidation is helpful in retarding LDL glycation. Hence, the probable mechanism by which *P. auritum* exerts its protective action is by minimizing the effects of free radicals, its antioxidant activity in association with the inhibition of lipid peroxidation.

### 2.5. Anti-AGES Activity Assay *in Vivo*

#### Effect of HS on Renal Glucose, Mitochondrial TBA-reactive Substance, Renal Weight, and AGE Levels

Kidney weight, renal AGEs and mitochondrial thiobarbituric acid-reactive substance was very elevated in diabetic rats compared to the control group (Table 7). These levels were reduced to almost in range values by the administration of the different isolated. The mitochondrial thiobarbituric acid-reactive substance was increased to 2.75 mmol/mg protein compared with the 1.85 mmol/mg protein of the control rats. These levels were equally decreased by the administration of HS and of aminoguanidine; additionally, the effect observed in the 200 mg/Kg of HS-treated group was the same as in the aminoguanidine-treated group. The level of glucose in the diabetic control group increased during the period of the experiment and the administration of the extract did not have an effect on it. Symptoms in diabetic animals are increased kidney lipid peroxidation (TBARS), reduction in antioxidant defense and increase in renal AGE. This was in agreement with the present study results that confirmed by Maillard-type fluorescent measurement, renal AGEs accumulation in streptozotocin-induced diabetic rats. For this reasons, we first assessed the effect of isolated on renal AGE accumulation and thiobarbituric acid reactive substance.

**Table 7:** Effect of HS on kidney contents of TBA-reactive substance, glucose, AGEs and renal weight.

Groups	TBA-reactive substance (mmol/mg protein)	Renal Weight (g)	AGE (AU)	Renal glucose (mg/g wet tissue)
Normoglucemic	1.85 ± 0.043 ^a^	0.75 ± 0.074 ^a^	16.03 ± 2.19 ^a^	0.81 ± 0.004 ^a^
Diabetic	2.75 ± 0.012	1.09 ± 0.065	24.25 ± 3.28	6.49 ± 1.67
HS	1.92 ± 0.082 ^a^	0.96 ± 0.012 ^a^	14.82 ± 2.38 ^a^	4.43 ± 1.36 ^a^
Aminoguanidine	1.87 ± 0.065 ^a^	0.94 ± 0.048 ^a^	12.87 ± 1.74 ^a^	4.10 ± 1.07 ^a^

Data as expressed as Mean ± SD; ^a^ *p* < 0.05 *vs*. diabetic control values.

Therefore, we first demonstrated renal AGE accumulation and the mitochondrial lipid peroxidation level. As a result, diabetic control rats showed significantly increased kidney weight and AGE accumulation, indicating renal hypertrophy, and also showed an increased level of TBA-reactive substance. Oral administration of HS ameliorated these changes. Particularly, HS successfully reduced AGE and TBA-reactive substance level at the dose of 200 mg/kg suggesting that *P.* *auritum* suppressed the state of oxidative stress, and decreased the levels of serum protein and hemoglobin glycosylated significantly, suggesting that it would inhibit oxidative damage and irreversible renal damage caused by the protein glycation reaction under diabetes conditions.

Diabetes mellitus is a serious metabolic disorder with micro and macrovascular complications that results in significant morbidity and mortality. Lipid peroxidation has been implicated in the pathogenesis of naturally occurring or induced diabetes [22]. Additionally, diabetes not only showed an increase in serum glycosylated protein, a parameter of Amadori products, e.g., glycosylated hemoglobin (HbA_1c_), produced in the glycation reaction, but also abnormal lipid metabolism, as shown by increased levels of total cholesterol, triglycerides, and TBA-reactive substance, a parameter of lipid peroxidation. Prolonged exposure to hyperglycemia in diabetes participates in the formation and accumulation of AGES which correlates with the severity of renal complications in diabetes [23]. This accumulation leads to an increase level of TBA-reactive substances.

Oxidative stress has been regarded as an important factor linking hyperlipidaemia with cardiovascular diseases. Normally, free radical production and antioxidant defence system are in a dynamic balance. When the balance is broken, oxidative stress occurs. Free radicals are formed disproportionately in diabetes by glucose oxidation, non-enzymatic glycation of proteins, and the subsequent oxidative degradation of glycated proteins. Abnormally high levels of free radicals and the simultaneous decline of antioxidant defense mechanisms can lead to damage of cellular organelles and enzymes, increased lipid peroxidation, and development of insulin resistance [24]. It was reported that high fat diets led to excess lipid accumulation in cells, which were an important causative factor for oxidative stress. When blood lipids are elevated, excessive reactive oxygen species were produced which would cause lipid peroxidation, cell membrane damage and lesions [25]. 

There is a wide range of antioxidant defenses which protect against the adverse effects of free radicals production *in vivo* [26]. Disturbances of antioxidant defense systems in diabetes mellitus have been reported [27]. Therefore, treatment with antioxidants may contribute to the prevention and delaying of diabetic complications [28].

Dietary measures form an important part of antidiabetic treatment. Leaves of *P. auritum* are used in daily food preparation in the southern states of Mexico. Our results in this experiment showed *P. auritum* has a significant antioxidant, metal chelation, free radical scavenging and anti-oxidative stress properties, has ability to reduce lipid peroxidation, formation of AGEs, has beneficial effects on renal metabolic abnormalities, including renal glucose, and AGEs, which are considered to play important roles in the development of diabetes complications. Therefore, this plant may have relevance in the prevention and treatment of diseases in which oxidants or free radicals or AGEs are implicated. In addition chemical studies are now being undertaken to characterize this plant’s bioactive substances.

## 3. Experimental

### 3.1. Plant Material and Preparation of Extracts

Fresh plants of *P. auritum* were collected in Mexico State. A voucher specimen (No. 7345) was deposited in the Herbarium of the UAM-Xochimilco, for further reference. Aerial parts were dried at room temperature and powdered (300 g). The powdered material was extracted consecutively with 900 mL of hexane, chloroform and methanol using a Soxhlet apparatus. These extracts was filtered and concentrated by rotary vacuum evaporator and kept in a vacuum desiccator for complete removal of solvent.

### 3.2. Estimation of Total Phenolic Content

Total soluble phenolic of the extracts were determined with Folin-Ciocalteu reagent using gallic acid as the standard [29].

### 3.3. Antioxidant Activity *in Vitro*

#### 3.3.1. 1,1-Diphenyl-2-picrylhydrazyl (DPPH) Assay

The ability of the extract to scavenge DPPH radicals was assessed as described by Gyamfi *et al.* [30]. This is one of the most extensively used antioxidant assays for plant samples. This method is based on scavenging of the DPPH radicals by antioxidants, which produces a decrease in absorbance at 517 nm. When a solution of DPPH is mixed with a substance that can donate a hydrogen atom, the reduced form of the radical is generated, accompanied by loss of color. This delocalization is also responsible for the deep violet color, characterized by an absorption band in ethanol solution at about 517 nm. An aliquot (50 µL) of extract or control was mixed with PBS (450 µL, 10 mM/L, pH 7.4) and methanolic DPPH (1.0 mL, 0.1 mM/L) solution. After a 30 min reaction, the absorbance was recorded at 517 nm on Perkin Elmer Lambda 11 spectrophotometer.

#### 3.3.2. Trolox Equivalent Antioxidant Capacity (TEAC) Assay

The antiradical properties of the extracts were determined using the TEAC assay. The TEAC assay is based on the reduction of the 2,2'-azinobis(3-ethylbenzothiazoline-6-sulfonic acid (ABTS) radical cation by antioxidants and was adapted with minor modifications from [31]. ABTS^+.^ radical cation was prepared by mixing ABTS stock solution (7 mM in water) with 2.45 mM potassium persulfate (K_2_S_2_O_8_). This mixture was left for 12–24 h in the dark until the reaction was complete and the absorbance was stable [Abs_734nm _to 0.700 (± 0.030)]. Plant extracts (1 mL), were allowed to react with the ABTS solution (1 mL) and the absorbance was taken at 734 nm after 7 min using a spectrophotometer (on Perkin Elmer Lambda 11 spectrophotometer). Appropriate solvent blanks were run in each assay. The scavenging capacity of the extract was compared with that of α-tocoferol and percentage inhibition calculated as ABTS radical scavenging activity:

(%) = [(Abs_control_ − Abs_sample_) /( Abs_control_)] × 100

where Abs_control_ is the absorbance of ABTS radical + methanol; Abs_sample_ is the absorbance of ABTS radical + sample extract/standard.

#### 3.3.3. Oxygen Radical Absorbance Capacity (ORAC) Assay

The assay was performed as per the method described by Cao *et al.* [32]. The reaction was carried out in 75 mM phosphate buffer (pH 7.4), and the final reaction mixture was 200 μL. The extracts at different concentrations and sodium fluorescein (67 μM) solutions were added in the well of the microplate. The mixture was preincubated for 10 min at 37 °C. AAPH (12 mM) was added rapidly using a multichannel pipette. The microplate was immediately placed in the reader, and the fluorescence kinetics was measured at 485 nm excitation and 520 nm emission wavelengths for 90 min. A blank using phosphate buffer instead of the antioxidant solution and five calibration solutions using Trolox as antioxidant were al so carried out in each assay. Sample curves (fluorescence versus time) were first normalized to the curve of the blank corresponding to the same assay. From the normalized curves, the area under the fluorescence decay curve (AUC) was calculated. The net AUC corresponding to a sample and the regression equations between net AUC and sample concentration were calculated. ORAC values were expressed as μmoles of Trolox equivalent with mM/g. The normalized area under curve (AUC) was calculated as:

AUC = *(fo* + *f_1_* + *f_2_* +… + *f_n_)/fo*

where *f*o is the initial fluorescence reading at 0 min and *f*k is the fluorescence reading at time *k* and *n* is the total number of time steps. The AUC net was obtained by subtracting the AUC of the blank from that of the sample or standard:

AUC_net _= AUC_sample/standard_ − AUC_blank_

#### 3.3.4. Ferrous Ion Chelating Ability

The method of Decker and Welch was used to investigate the ferrous ion chelating ability of extracts [33]. The ferrous chelating ability of the fractions was monitored by measuring the formation of the ferrous ion ferrozine complex. The reaction mixture containing 1.0 mL of different concentrations of the extracts (50–800 µg/mL) was mixed with methanol (3.7 mL), 2 mM ferrous chloride (0.1 mL) and 5 mM ferrozine (0.2 mL) to initiate the reaction and the mixture was shaken vigorously and left to stand at room temperature for 10 min. The absorbance of the solution was measured at 562 nm. The percentage chelating effect on ferrozine-Fe^2+^ complex was calculated. The % values were compared with ascorbic acid. 

#### 3.3.5. Nitric Oxide Radical Scavenging Assay

This assay was performed according to the method described by Sreejayan *et al*. [25]. Nitric oxide generated from sodium nitroprusside in aqueous solution at physiological pH interacts with oxygen to nitrite ions, which was measured by Griess reagent. The reaction mixture (3 mL) containing 10 mM nitroprusside in phosphate buffered saline, and the fractions or the extracts at different concentrations (50–800 µg/mL) were incubated at 25 °C for 150 min. An aliquot (about 0.5 mL) of incubated sample was removed at 30 min intervals and 0.5 mL Griess reagent was added. The absorbance of chromophore formed was measured at 546 nm. Inhibition of the Nitric oxide generated was measured by comparing the absorbance values of control and extracts.

#### 3.3.6. β-Carotene Bleaching (BCB) Assay

A solution of β-carotene was prepared by dissolving β-carotene (2 mg) in chloroform (10 mL) and this solution (1.0 mL) was then pipetted out into a flask containing linoleic acid (20 mg) and Tween 40 emulsifier (200 mg). Chloroform was completely evaporated using a vacuum evaporator. Aliquots of 5.0 mL of this emulsion were transferred into a series of tubes containing various concentrations of the fractions (25–400 µg/mL) or tocopherol. The absorbance of the extracts and the standard was measured immediately (t = 0) and after 90 min at 470 nm. The tubes were incubated at 50 °C in a water bath during the test. The antioxidant activities (AA) of the samples were evaluated in terms of bleaching of β-carotene using the following formula: 

AA = 100[1 − A0 − At)/Ao * − At *)]

where A_0_ and A_0_ * are the absorbance values measured at zero time of incubation for test sample and control respectively and A_t_ and A_t_ * are the absorbance values of the test sample and control respectively, after incubation for 90 min [26].

#### 3.3.7. Thiocyanate Method

The peroxy radical scavenging activity was determined by thiocyanate method using α-tocopherol (50–800 µg/mL) as standard [27]. Increasing concentration of the fractions (50–800 µg/mL) in 0.5 mL of distilled water was mixed with 0.02 M linoleic acid emulsion (2.5 mL, in 0.04 M phosphate buffer pH 7.0) and phosphate buffer (2 mL, 0.04 M, pH 7) in a test tube and incubated in darkness at 37 °C. At intervals during incubation, the amount of peroxide formed was determined by reading the absorbance of the red colour developed at 500 nm by the addition of 30% ammonium thiocyanate solution (0.1 mL) and 20 mM ferrous chloride in 3.5% hydrochloric acid (0.1 mL) to the reaction mixture. The percentage scavenging activity was calculated and the values of the extracts were compared with the standard, α-tocopherol.

#### 3.3.8. Superoxide Radical Scavenging Assay

The scavenging activity of the crude extract against chemically generated superoxide radicals (O_2_·**^−^**) was measured by means of spectrophotometric measurement of the product obtained on reduction of nitroblue tetrazolium (NBT). Superoxide anions were generated in a nonenzymatic PMS/NADH system [28]. The reaction mixture contained test solution (1 mL), 0.1 M phosphate buffer (1.9 mL, pH 7.4), 20 µM phenazine methosulfate (PMS, 1 mL), 156 µM nicotine adenine dinucleotide (NADH), and 25 µM NBT in phosphate buffer (pH 7.4). Alter 2 min of incubation at 25 °C, the color was read on a spectrophotometer at 560 nm against blank samples that contained no PMS.

### 3.4. Antioxidant Activity Assay *in Vivo*

#### 3.4.1. Animals

The study was conducted in male mice, weighing about 20–25 g. Before and during the experiment, animals were fed a standard laboratory diet (Mouse Chow 5015, Purina) with free access to water. Mice were procured from the bioterium of ENCB and were housed in cages under standard laboratory conditions (temperature 25 ± 2 °C). Animals were acclimatized for a period of three days in their new environment before the initiation of experiments. Litter in cages was renewed three times a week to ensure hygiene and maximum comfort for animals. The experiments reported in this study were can out following the guidelines stated in Principles of Laboratory Animal Care (NIH publication 85-23, revised 1985 and the Mexican Official Norms, Norma Oficial Mexicana NOM-062-Z00-1999). Food consumption and weight gain were measured daily. 

#### 3.4.2. Streptozotocin (STZ)-Induced Diabetic Model

Severe diabetes mellitus was induced in overnight fasted male rats by a single intraperitoneal injection of streptozotocin dissolved in cold citrate buffer (pH 4.5), at a dose of 50 mg/kg body weight [29]. The rats with diabetic symptoms such as polydipsia and polyuria, as well as, fasting blood glucose concentration higher than 13 mmol/L after 7 days of STZ injection were selected for use as experimental animals. In some cases, a STZ injection may trigger massive insulin release and result in fatal hypoglycemia. This hypoglycemic period was followed by hyperglycemia which then became permanent. To prevent death induced by STZ, the rats were fed with a 3% glucose solution for 24 h. 

#### 3.4.3. Experimental Design

In the experiment a total 24 rats for each extract were divided into seven groups (n = 4 per group): normal control (group 1), diabetes control (group 2), and diabetic rats treated with *P. auritum* hexane extract at doses of 400 mg/kg body weight on a daily basis for 28 days (group 3). Group 4 were diabetic rats treated with glibenclamide at 0.5 mg/kg. All the drugs solutions or vehicle were administered orally by gastric intubations once daily at 9:00 a.m. for 30 days. At the end of the experiment rats were sacrificed by cervical dislocation and liver, kidney and pancreas of each rat was removed, cleaned of fatty tissue. Lipid peroxidation, that is, thiobarbituric acid reactive substances (TBARS) was estimated by the method of Fraga *et al*. [30] and expressed as µM/g of liver, pancreas and kidney tissue.

#### 3.4.4. Measurements of GSH, SOD, CAT and GPx

Antioxidant enzyme activities in the liver and kidney were assayed using commercial kits: superoxide dismutase (SOD) assay kit Bioxytech SOD-525 for SOD activity (Oxis International, Beverly Hills, CA, USA), catalase assay kit for catalase activity (CAT) (Cayman Chemical, Ann Arbor, MI, USA), and glutathione reductase (GSH) assay kit Bioxytech GR-340 for GR activity, (Oxis International) and glutathione peroxidase (GPx) assay kit GPx-340 for GPx (Oxis International).

#### 3.4.5. Statistical Analysis

All experiments were performed in triplicate (n = 3) and results were expressed as mean ± SEM. Statistical analysis was carried out by using OriginPro 7.5 software. One way ANOVA was applied to data and results were compared by using Tukey test. A difference was considered to be statistically significant when the p-value is lower than (*p* < 0.05). 

### 3.5. Anti-AGES Activity Assay *in Vitro*

#### 3.5.1. Bovine Serum Albumin (BSA)-Glucose Assay

The methodology was based on that of Brownlee *et al.* [31]. BSA (l0 mg/mL) was incubated with glucose (500 mM) in phosphate buffered-saline (PBS) (5 mL total volume, pH 7.4) and extract containing 0.02% sodium azide at 37 °C. All the reagent and samples were sterilized by filtration through 0.2 µm membrane filters. The protein, the sugar and the prospective inhibitor were included in the mixture simultaneously. Aminoguanidine was used as an inhibitor positive control. Reactions without any inhibitor were also set up. Each solution was kept in the dark in a capped tube. After 15 days of incubation, fluorescence intensity (excitation wavelength of 370 nm and emission wave-length of 440 nm) was measured for the test solutions. Percent inhibition was calculated as follows:

Inhibition %= [1 − (As − Ab)/(Ac − Ab)] × 100

where As = fluorescence of the incubated mixture with sample, A_c_, A_b_ = are the fluorescence of the incubated mixture without sample as a positive control and the fluorescence of incubated mixture without sample as a blank control.

#### 3.5.2. BSA-Methylglyoxal Assay

This assay was modified based on a published method [32]. The assay evaluates the middle stage of protein glycation. BSA and methylglyoxal were dissolved in phosphate buffer (100 mM, pH 7.4) to a concentration of 20 mg/mL and 60 mM, respectively. Extract or fractions were dissolved in the same phosphate buffer. One milliliter of the BSA solution was mixed with methylglyoxal solution (1 mL) and OM extract (1 mL). The mixture was incubated at 37 °C. Sodium azide (0.2 g/L) was used as an aseptic agent. Phosphate buffer was used as a blank. Aminoguanidine and phloroglucinol were used as positive controls. After seven days of incubation, fluorescence of the samples was measured using an excitation of 340 nm and an emission of 420 nm, respectively:

The % inhibition of AGE formation = [1 − (fluorescence of the test group/fluorescence of the control group)] × 100%.

#### 3.5.3. Glycation of Hemoglobin

Glassware was previously sterilized. The experiment was performed on a specially treated bench to avoid any possible contamination. Glucose (0.278 mg/dL), hemoglobin (30 mg/dL), extract (5 mg/dL), and glutathione (0.5 mM) were dissolved in distilled-sterilized water. This hemoglobin solution was diluted with three times as much water for the negative control group. Hemoglobin, glucose, and water were mixed at a ratio of 1:1:2 (v/v/v). Isolated or glutathione was added instead of water to the positive control group. These mixtures were incubated for 5 days at 37 °C with continuous stirring (70 rpm). The amount of glycated hemoglobin (% GHb) was determined using an ion capture component set (IMx system, Abbott laboratories, USA). The amount of hemoglobin A_lc_ (%HbA_lc_) was calculated by defining the equation used to convert IMx glycated hemoglobin (% GHb) to standardized percent of hemoglobin A_lc _(% HbA_lc_). 

#### 3.5.4. LDL Oxidation Measurement

Amphotericin B was dissolved in methanol first and then added to an LDL solution, with or without compound treatment, for final concentration of 5 and 10 µM of amphotericin B. One milliliter of CuSO_4_ (10 µM) was used to initiate LDL oxidation in an LDL solution sample (10 mL). After incubating the LDL solution at 37 °C for 72 h, the method of Jain and Palmer [33] was used to measure malondialdehyde (MDA) formation (nmol/mg LDL protein). Briefly, LDL solution (0.2 mL) was suspended in PBS (0.8 mL). Then trichloroacetic acid (TCA; 0.5 mL, 30%) was added. After vortexing and standing in ice for 2 h, samples were centrifuged at 1,500 × *g* for 15 min. Supematant (1 mL) was mixed with thiobarbituric acid (TBA; 0.25 mL, 1%), and the mixture was heated in a boiling water bath for 15 min. The concentration of MDA-TBA complex was assayed with at 532 nm. 

The formation of conjugated diene (CD), a lipid oxidation product, in LDL also was determined according to the method described by Esterbauer *et al.* [34]. The lipid oxidation of an LDL solution containing 5 or 10 µM of each compound was initiated at 37 °C by 0.1 mM CuC1_2_. Absorbance at 234 nm was continuously recorded for 60 min at 37 °C by a Hitachi U-2001 spectrometer with a constant temperature recirculate. The lag phase, expressed in minutes, was defined as the period where no oxidation occurred. A longer lag phase indicated less CD formation. 

#### 3.5.5. *In Vitro* Glycation of LDL

LDL glycation was performed according to the method described in Li *et al.* [35]. Briefly, 50 mM glucose in PBS (pH 7.4) was added to an LDL solution with and without compound treatment. Sodium azide at 0.02% was used as antibiotic to prevent bacterial growth. This solution was sterile filtered, covered with N_2_, and stored for 6 d at 37 °C in the dark. After glycation, the solutions were dialyzed against PBS (20 mL, against 4 L) at 4 °C for 40 h. Then glycated LDL was separated from nonglycated LDL by applying a GlycoGel II column (Pierce, Rockford, IL, USA), in which 500 µL LDL solution was loaded on the column, and glycated LDL was eluted with 2 mL, sorbitol buffer, pH 10.25. Neither copper nor any other oxidant was used for the experiments on LDL glycation. The method of Duell *et al.* [36] was used to measure LDL glycation level. LDL solution (200 µL) was mixed with 4% NaHCO_3_ (200 µL) and 0.1% trinitrobenzoic acid (200 µL). This mixture was flushed with N_2_, sealed, and incubated at 37 °C in the dark. After 2 h, the absorbance at 340 nm was measured spectrophotometrically. The blank was a mixture of LDL and NaHCO_3_ in PBS. LDL glycation is reported as relative reduction in the level of free ε-amino groups of L-lysine when compared with LDL solution in the absence of glucose. During LDL glycation, samples were treated with or without EDTA (0.5 mM), and LDL oxidation level was also determined. 

### 3.6. Anti-AGES Activity Assay *in Vivo*

#### Mitochondrial TBA-Reactive Substance Level and AGE Level in Kidney

Mitochondria were prepared from kidney homogenate by differential centrifugation (800 × g and 12,000 × g, respectively) at 4 °C according to the methods of Jung and Pergande [37], with minor modifications. Each pellet was resuspended in preparation medium and the concentration of TBA-reactive substance was determined by the method of Mihara and Uchiyama [32]. The renal AGE level was determined by the method of Nakagawa [16]. In brief, minced kidney tissue was treated with chloroform and methanol (2:1, v/v) overnight. After washing, the tissue was homogenized in 0.1 N NaOH, followed by centrifugation at 8,000 × g for 15 min at 4 °C. The amounts of AGEs in these alkali-soluble samples were determined by measuring the fluorescence at an emission wavelength of 440 nm and an excitation wave length of 370 nm. A native BSA preparation (l mg/mL of 0.1 N NaOH) was used as a standard, and its fluorescence intensity was defined as one unit of fluorescence. The fluorescence values of samples were measured at a protein concentration of 1 mg/mL and expressed in arbitrary units (AU).

## 4. Conclusions

In conclusion, the study demonstrated that leaves of *Piper auritum* had significant antioxidant captivities, and also possessed high antioxidant abilities. Using different *in vitro* and *in vivo* glycation models, hexane extract from *P. auritum* possesses a potent inhibitory effect on formation of AGEs which might be attributed to its antioxidative activity. The administration of *P. auritum* for 28 days to STZ-induced diabetic rats could ameliorate oxidative stress and the mitochondrial lipid peroxidation level in kidney and to improve lipid metabolism as well as to reduce the imbalance between the generation of ROS and the scavenging enzyme activity in diabetic conditions. Additional studies are needed to characterize the bioactive compounds responsible for the observed activities. This study might be helpful in the development of medicinal preparations or functional food for diabetes and related symptoms.
